# Prescription‑dose stratification improves deep learning‑based VMAT dose prediction in locally advanced NSCLC

**DOI:** 10.1038/s41598-026-43192-6

**Published:** 2026-03-09

**Authors:** Thitaporn Chaipanya, Kampheang Nimjaroen, Sasikarn Chamchod, Panatda Intanin, Patiparn Kummanee, Dhammathat Owasirikul, Chirasak Khamfongkhruea

**Affiliations:** 1 Medical Physics Program, Princess Srisavangavadhana Faculty of Medicine, Chulabhorn Royal Academy, 906 Kamphaeng Phet 6 Rd., Talat Bang Khen, Lak Si, Bangkok, 10210 Thailand; 2 Radiation Oncology Department, Chulabhorn Hospital, Chulabhorn Royal Academy, Bangkok, Thailand; 3 Bachelor of Science Program in Radiological Technology, Kanchanabhishek Institute of Medical and Public Health Technology, Nonthaburi, Thailand; 4 Personalized Radiotherapy and Imaging in Medicine (PRIME) Research Center, Chulabhorn Royal Academy, Bangkok, Thailand

**Keywords:** Dose prediction, Deep learning, Non-small cell lung cancer (NSCLC), 3D U-Net, Volumetric-modulated arc therapy (VMAT), Cancer, Diseases, Health care, Medical research, Oncology

## Abstract

Volumetric-modulated arc therapy (VMAT) planning for locally advanced non-small cell lung cancer (NSCLC) is an iterative and planner-dependent process that often requires multiple optimization cycles to balance target coverage and organ‑at‑risk (OAR) sparing. Deep‑learning dose prediction can accelerate planning by providing patient‑specific reference dose distributions, but the impact of prescription‑dose mixing during model training remains unclear. This study evaluated whether prescription‑stratified models improve VMAT dose prediction performance. Seventy-two NSCLC VMAT cases were recalculated to 50, 54, and 60 Gy and split into training, validation, and test sets (42/10/20 cases). Four models with identical 3D U-Net architecture were developed: three single-prescription models (50/54/60 Gy) and one mixed-prescription model (50 + 60 Gy). Performance was assessed using mean absolute error (MAE) for planning target volume (PTV) and OAR dose metrics. Single-prescription models reproduced PTV coverage (D_95%_ and D_99%_) with MAEs < 4 Gy and hot-spot (D_2cc_ and D_5cc_) errors < 1 Gy, while mean dose errors for lungs and heart were ≤ 2.3 Gy. The mixed-prescription model showed larger errors: PTV hot-spot MAE rose to 11.3 Gy, and spinal cord maximum-dose errors reached 5–6 Gy, although most other OAR metrics changed modestly. Voxel‑wise difference maps revealed local deviations of a few Gy in low-dose lung regions and near steep gradients. These findings indicate that prescription‑dose stratification improves clinically relevant prediction metrics and support deep‑learning dose prediction as a planning decision‑support and optimization‑guidance tool.

**Supplementary Information:**

The online version contains supplementary material available at 10.1038/s41598-026-43192-6.

## Introduction

Lung cancer is the second most common cancer and the leading cause of cancer-related mortality worldwide^1^, placing a significant burden on health systems. Non-small cell lung cancer (NSCLC) accounts for the majority of lung cancers and is therefore a major focus of curative‑intent treatment strategies^2–4^. For locally advanced NSCLC, complete surgical resection is often not feasible, and concurrent or sequential chemoradiation becomes the standard of care^5^. Among radiation‑therapy techniques, external beam radiotherapy with advanced modalities such as volumetric‑modulated arc therapy (VMAT) enables highly conformal dose delivery that maximizes tumor coverage while sparing normal tissues^6–8^. However, VMAT planning for large thoracic targets adjacent to critical organs (lungs, heart, spinal cord) remains complex and typically requires multiple rounds of manual, trial‑and‑error optimization, which increases planner workload and prolongs the overall planning process^9–12^, leading to a time-consuming optimization loop. Furthermore, if lung dose constraints—such as V_20Gy_ (the percentage of the total lung volume receiving ≥ 20 Gy)—are predicted to exceed tolerance limits, the radiation oncologist may consider reducing the prescribed total dose and/or adjusting the planning approach. If constraints remain unachievable despite these modifications, the treatment strategy may shift toward non-radiation management with chemotherapy alone. Therefore, early, anatomy-based dose prediction could provide a rapid estimate of achievable target coverage and organ-at-risk (OAR) dose volume histograms (DVHs) to screen plan feasibility (e.g., identify cases likely to violate lung constraints such as V_20Gy_) and reduce avoidable planning iterations.

Artificial intelligence (AI) is increasingly integrated into radiotherapy to improve treatment planning efficiency and consistency^13,14^, with dose prediction playing an important role as a clinical decision-support tool^15,16^. One of the earliest AI-driven approaches in this domain is knowledge-based planning (KBP)^17,18^—a technique that uses historical treatment plans to predict dose distributions and guide optimization. While early KBP was limited by scalar summaries that lost spatial information, modern KBP performs voxel-wise 3D dose prediction, as seen in the AAPM Open-KBP Grand Challenge^19^. However, these methods can still be constrained by hand-engineered geometric features that under-represent complex anatomy^10^. To overcome these limitations, deep learning (DL) has emerged as a recommended approach for radiation dose prediction^20–23^. DL models like Convolutional Neural Networks (CNNs) learn features directly from imaging data to produce voxel-level dose distributions without hand-engineered inputs^10,17,22,23^. This has driven rapid growth in DL-based automated treatment planning^11^, which has shown promising performance for dose prediction^17,22,23^ in several disease sites and can reduce manual effort and improve planning consistency and standardization, potentially freeing time for complex cases that require additional expert review.

While DL-based dose prediction for NSCLC is advancing, most prior work has often focused on fixed-field IMRT or has not systematically compared different modeling strategies for handling multiple prescription levels^24–26^. Important contributions include Shao et al.^27^, who proposed an asymmetric network for multi-prescription IMRT, Barragán-Montero et al.^23^’s beam-aware dense U-Net for 60 Gy IMRT, Zhang et al.^24^’s attention-augmented ResUNet for 60 Gy VMAT, and Cao et al.^25^’s multi-prescription 3D U-Net. Although mixed‑prescription models can perform well when prescription information is explicitly incorporated; however, the impact of training on combined prescriptions without prescription‑aware conditioning has not been systematically assessed for VMAT, where steep dose gradients and complex modulation may worsen domain shift. Consequently, a key implementation question therefore remains whether a single mixed‑prescription model can achieve VMAT dose‑prediction performance comparable to dedicated single‑prescription models for NSCLC, or whether prescription‑stratified models are required despite added complexity. In this study, four 3D U-Net models are developed and compared: three models stratified by prescription (50, 54, and 60 Gy) and one mixed-prescription model trained on 50 and 60 Gy cases. The aims are to determine whether prescription-dose stratification yields clinically meaningful improvements in VMAT dose prediction for locally advanced NSCLC and to define the role of such models as planning decision-support tools to enhance feasibility screening and optimization rather than to replace treatment-planning-system (TPS) dose calculation and verification.

## Materials and methods

**Fig. 1:**
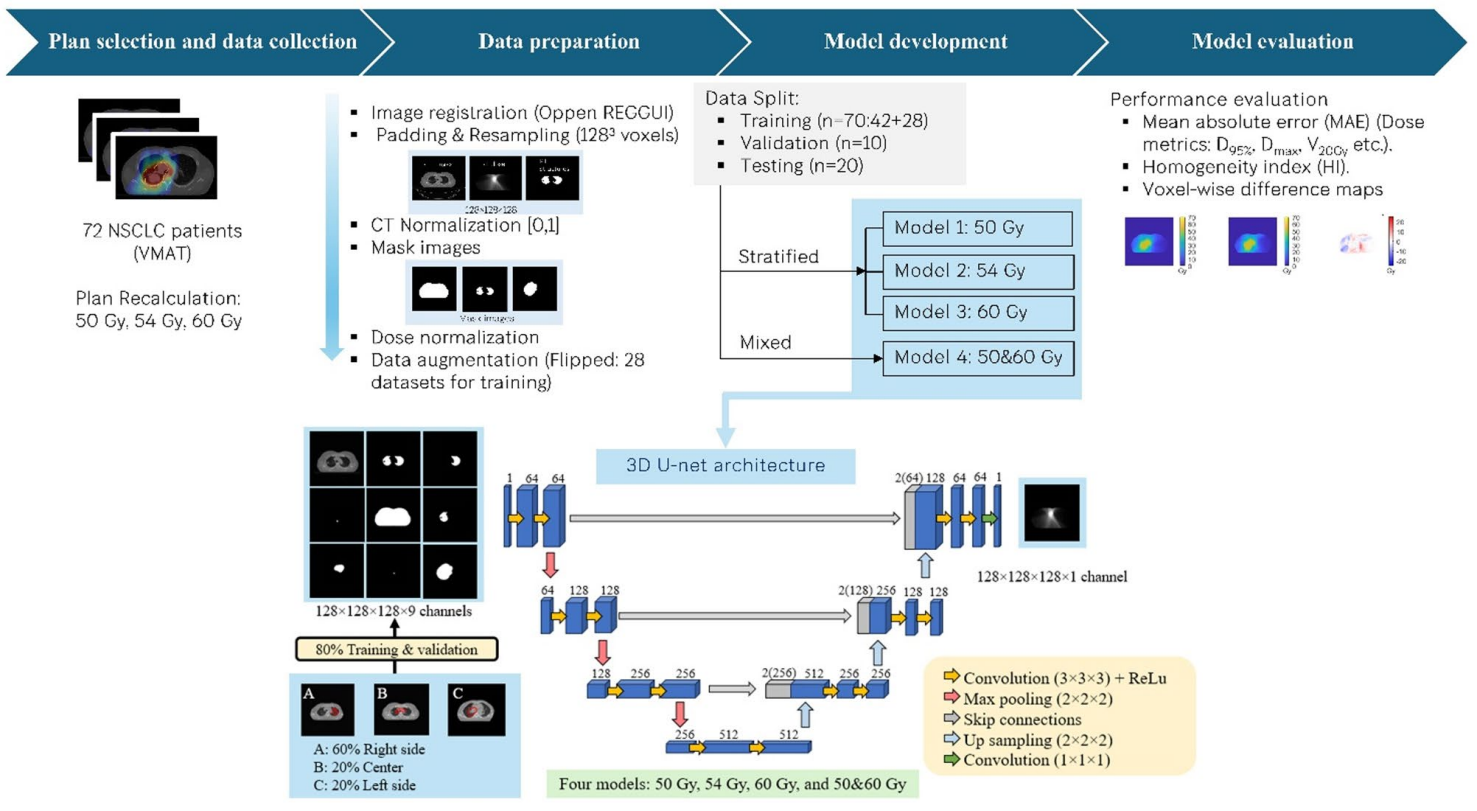
Integrated methodological workflow and 3D U-Net architecture for deep-learning–based VMAT dose prediction. 3D U-Net model with 128 × 128 × 128 voxels and nine input channels—one for the planning target volume (PTV), seven for organs-at-risk (OARs), and one for CT images—to predict the three-dimensional dose distribution. Each blue box represents a multi‑channel feature map, gray boxes show copied feature maps, and arrows denote the different operations.

A summary of the workflow used in this study is provided in Fig. 1, which outlines the main methodological steps, including VMAT plan recalculation at three prescription levels, data preprocessing to generate standardized 9-channel input volumes, dataset division for training, validation, and testing, development of prescription-specific and mixed DL models, the 3D U-Net architecture used for dose prediction, and the overall model evaluation process.

### Plan selection and data collection

In this study, we retrospectively analyzed 72 cases of NSCLC treated between 2015 and 2024 at the Radiation Oncology Department of Chulabhorn Hospital. In the original 72‑patient cohort, 23 patients were planned at 50 Gy, 9 at 54 Gy, and 40 at 60 Gy, reflecting the prescription patterns used in our clinical practice. Patient selection adhered strictly to the National Comprehensive Cancer Network (NCCN) guideline^2^, which advocate for chemoradiation therapy (sequential or concurrent) for NSCLC patients with positive margins across stages IIB–IIIAB. Preoperative CCRT is advised for resectable cases, while definitive CCRT is recommended for unresectable cases. All patients were simulated and treated in the supine position using our institutional thoracic immobilization protocol, with both arms raised on a wing board to ensure a stable and reproducible setup. This standardized immobilization approach was applied uniformly across all patients, thereby minimizing inter-patient variability related to setup and positioning during model training and evaluation. This study was approved by the Institutional Review Board of Chulabhorn Royal Academy under approval number EC 125/2566. Informed consent was obtained from all subjects and/or their legal guardians. The study was performed in accordance with the Declaration of Helsinki. Given the limited sample size, a structured data split was adopted to ensure model robustness and sufficient testing. The 72 original patient cases were randomly divided into a training set (42 cases), a validation set (10 cases), and a test set (20 cases). To expand the training data and improve the robustness of the model, we performed left–right flipping augmentation on 28 out of the 42 training cases. These 28 cases were selected using simple random sampling without replacement, stratified to maintain the original tumor‑location distribution (60% right lung, 20% left lung, 20% central). This procedure yielded a total of 70 unique anatomical–dose paired training datasets, consisting of 42 original and 28 augmented cases, while the validation (10 cases) and independent test set (20 cases) remained unaugmented. This design was chosen to guarantee patient‑level independence between training, validation, and test sets, so that no patient appears in more than one split, at the cost of relying more heavily on augmentation within the training set. We acknowledge that this strategy may limit the diversity of non‑mirrored anatomies available for training and is further considered in the Discussion. For the mixed-prescription model, the 50 Gy and 60 Gy cohorts were fully combined prior to data splitting. This resulted in 140 training samples (70 at 50 Gy and 70 at 60 Gy). The validation set comprised 20 samples (10 cases × 2 prescriptions), and the independent test set comprised 40 samples (20 cases × 2 prescriptions), which were used exclusively for model selection and final evaluation.

Treatment planning was standardized throughout the study. Contouring followed NCCN-aligned institutional protocols, which were applied consistently across the study period. All patients were treated with VMAT. For model development, datasets were grouped according to three prescription doses—50 Gy, 54 Gy, and 60 Gy (all delivered at 2 Gy per fraction). Treatment plans employed asymmetric avoidance arcs (40°–120° and 300°–240°), tailored to patient anatomy and designed to reduce low-dose lung exposure in accordance with institutional protocol. This asymmetric avoidance-arc configuration reflects institution-specific clinical practice. While it was applied consistently across all cases to ensure internal consistency for model training and comparison, different VMAT arc strategies (e.g., full arcs with partial blocking or contralateral lung–avoidance arcs) may produce different dose patterns and modulation characteristics. As a result, the generalizability of the trained models to centers using alternative arc arrangements may be limited, and external validation or retraining using institution-specific planning protocols would be required prior to clinical deployment. Dose distributions were normalized to the PTV D_95%_ (the dose covering 95% of the PTV). All treatments used 6 MV photon beams and followed NCCN dose constraints^2^. Calculations were performed using the Anisotropic Analytical Algorithm (AAA) algorithm in Eclipse TPS v16.1 (Varian Medical Systems, Palo Alto, CA, USA). All dose distributions were calculated with a 2.5 mm grid resolution. The uniform grid size across all cases ensures consistency in spatial resolution, which is critical for training DL models. Variations in grid size could introduce artifacts or inconsistencies in the learned dose patterns; therefore, maintaining a standardized grid resolution was essential for model reliability. CT images, contours, and dose distributions were exported as DICOM files for model development.

### Data preparation

Each patient’s data was co-registered using the Registration Graphical User Interface (OpenREGGUI), a MATLAB-based tool, to align CT images, contours, and dose distributions at 1 mm per pixel resolution. This alignment resulted in 512 × 512 × Z, where Z (the number of slices) varied per patient (mean: 110, range: 85–142). As the number of slices (z) varied across cases, we first applied zero padding (i.e., adding boundary voxels with value 0 outside the patient) to standardize the volume size (preserve the original aspect ratio) and then resized all channels to 128 × 128 × 128 voxels. Given that thoracic CTs may contain lung-related artifacts (e.g., respiratory motion and streaking), we applied HU clipping and intensity normalization; the CT images were then normalized using the rescaling method described in Eq. 1 to adjust pixel values within a range of 0 to 1, thereby ensuring balanced feature contribution in DL models; cases with substantial metal implants were excluded, and robustness to severe metal artifacts was not assessed.

1 $$X_{{Rescaled}} = \frac{{\left( {X_{{Original}} - X_{{Min}} } \right)}}{{\left( {X_{{Max}} - X_{{Min}} } \right)}}$$

where X_Original_ represents the intensity of a given pixel in the original CT image, and X_Min_ and X_Max_ are the minimum and maximum pixel intensities found within that image, respectively. This transformation linearly maps all original CT values into the [0,1] range, preserving the relative contrast while standardizing the intensity scale.

To focus on relevant anatomical areas, non-essential regions were cropped, while masks for essential structures, including the PTV, esophagus, heart, lungs, spinal cord, and body, were extracted from the DICOM-RT structure sets. These masks were then converted into separate binary channels to serve as input data for model training and testing. The dose information extracted from the DICOM files was first converted to Gy units and normalized to the corresponding prescription dose. To create a consistent multi-prescription dataset, each patient’s plan was then recalculated at all three prescription levels (50 Gy, 54 Gy, and 60 Gy) using the same beam geometry and contours, with dose renormalization to PTV D_95%_, without re-optimization. This resulted in three standardized dose distributions per patient and ensured uniform representation across all prescription categories.

### Model development

For model development, CT images and patient contours were used as input data, while the corresponding 3D dose distributions served as the ground truth. All input data—including CT images, structure masks, and dose distributions—were resampled to a uniform voxel spacing of 2.5 × 2.5 × 2.5 mm³ using trilinear interpolation to ensure consistency across patients. The resampled volumes were then standardized to a fixed size of 128 × 128 × 128 voxels, a dimension selected based on GPU memory limitations commonly encountered in 3D deep learning. This standardization ensures that spatial features are represented at consistent scales, which is essential for stable model training and performance. The models were developed using the 3D U-Net architecture, a common and effective choice for DL-based dose prediction due to its capacity to capture detailed spatial information through an encoder-decoder structure (Fig. 1). Our implementation utilized nine distinct input channels: one for the PTV, seven for organs-at-risk (OARs), and one for the CT imaging data. All nine input channels are three-dimensional, co-registered volumes (CT as a 3D image volume and each structure as a 3D binary mask volume) stacked to form a 9 × 128 × 128 × 128 input tensor. The input to the network was a 9-channel tensor, where each channel represented a binary mask for the PTV, an OAR (esophagus, heart, left lung, right lung, spinal cord, body), or the normalized CT image. All four models used the same 3D U-Net architecture, training pipeline, and nine anatomy-based input channels, optimized with an MAE loss. The prescription dose was deliberately excluded from both the inputs and the loss, to specifically assess the effect of training on combined prescriptions without explicit prescription conditioning. The 3D U-Net was implemented in MATLAB R2023b (MathWorks, Natick, MA, USA) using the Deep Learning Toolbox, with all components programmed in-house following the standard 3D U-Net architecture. The training was performed on a high-performance Lenovo ThinkVision workstation equipped with an Intel Xeon Gold 6258R processor and NVIDIA Quadro RTXP8000 GPU. A single data split was used for training and evaluation. The models were trained using the Adaptive Moment Estimation (Adam) optimizer and a Mean Absolute Error (MAE) loss function. The training configuration included a batch size of 4, an initial learning rate of 0.0001, and up to 500 training epochs. To prevent overfitting, early stopping was implemented based on the validation set performance; the model weights that yielded the lowest validation loss were selected for the final model. Importantly, all four models shared the same network architecture and training settings; they differed only in the prescription composition of the training data (single‑prescription: 50/54/60 Gy; mixed‑prescription: 50 + 60 Gy).

This study developed four separate models: three models correspond to individual dose prescriptions of 50 Gy, 54 Gy, and 60 Gy, respectively, while the fourth is a mixed-prescription model trained on both 50 Gy and 60 Gy cases. The purpose of the mixed-prescription model was to evaluate the model’s generalizability across different prescription levels. For this model, the 50 Gy and 60 Gy plan variations from the 42 training patients were used, creating a larger training set of unique anatomy-dose pairings. The mixed-prescription model was trained and evaluated exclusively on 50 Gy and 60 Gy cases. Intermediate prescription levels (e.g., 54 Gy) were not included in either training or testing and were therefore not used to assess interpolation across prescription doses.

### Model evaluation

To assess model performance on unseen data, 20% of the dataset (20 cases) was reserved as an independent test set and was not used during training or validation. Model performance was evaluated using two complementary approaches: quantitative DVH metrics and dose distribution analysis. Quantitative evaluation used MAE for PTV and OAR DVH endpoints (Table 1), including homogeneity index (HI) as defined in Eqs. 2 and 3.

### Quantitative DVH metrics

This evaluation involved comparing predicted dose distributions with ground truth values using mean absolute error (MAE) for DVH metrics, as specified in Table 1, and homogeneity index (HI), as outlined in Eqs. 2 and 3, respectively.

- MAE: Computed for all DVH-derived metrics (e.g., D_99%_, D_mean_, V_95%_, D_2cc_) by averaging the absolute errors across the N patients in the test set:

2 $${\text{MAE }} = \frac{1}{N}\mathop \sum \limits_{{i = 1}}^{N} \left| {{\mathrm{p}}_{{{\mathrm{Ref}},{\mathrm{i}}}} - {\mathrm{p}}_{{{\mathrm{Pre}},{\mathrm{i}}}} } \right|$$

where p_Ref, i_ and p_Pre, i_ are the metric values from the ground truth and prediction for the i_th_ patient, respectively.

- HI: Applied exclusively to the PTV to evaluate dose uniformity:

3 $${\text{HI }} = \frac{{D_{{5\% }} }}{{D_{{95\% }} }}$$

where D_5%_ and D_95%_ denote the doses delivered to 5% and 95% of the PTV, respectively, a value closer to 1 indicates more homogeneous dose coverage.

All statistical tests were performed on paired per-patient DVH metrics comparing predicted and reference values within the independent test set. The Shapiro-Wilk test was used to assess data normality. A t-test was subsequently applied to normally distributed data, while the Wilcoxon signed-rank test was used for data that did not follow a normal distribution.

**Table 1:** Dosimetric evaluation parameters for planning target volume (PTV) and organs-at-risk (OAR).

Organ	Criteria
PTV	D_99%,_ D_98%,_ D_95%,_ D_mean,_ D_2cc,_ D_5cc_
	V_95%_
	HI
Spinal cord	D_max,_ D_2cc_
Lung	D_mean_, V_5Gy_, V_20Gy_
Heart	D_mean_, D_2cc_, V_30Gy_, V_35Gy_, V_40Gy_
Esophagus	D_mean_, D_2cc_, V_40Gy_, V_50Gy_

### Dose distribution and DVH-based assessment

For the dose distribution and DVH-based assessment, axial dose distributions were overlaid (prediction vs. ground truth), and pixel-wise difference maps were generated to calculate spatial deviations between the prediction and ground truth, with a particular focus on both high- and low-dose regions. In parallel, DVH from the predicted and reference were plotted together for PTV and OARs. We specifically analyzed high- and low-dose regions, as well as steep gradients, to detect any systematic overestimation or underestimation.

## Results

### Prediction accuracy for PTV metrics

MAEs for the PTV are summarized in Table 2. Overall, the eight dosimetric parameters evaluated (D_99%_, D_98%_, D_95%_, D_mean_, D_2cc_, D_5cc_, V_95%_, and HI), the single-prescription models (Models 1–3) consistently exhibited lower MAEs than the mixed-prescription model (Model 4). For the high-dose coverage indices (D_99%_, D_98%_, D_95%_), the prescription-stratified models consistently outperformed the mixed-prescription model. The 50 Gy model achieved the lowest errors across coverage metrics, while the 60 Gy model showed slightly higher variability, with statistically significant deviations for D_99%_. In contrast, the mixed-prescription model demonstrated significantly larger errors across all high-dose coverage indices. Errors in mean and hot-spot dose metrics (D_mean_, D_2cc_, and D_5cc_) remained low for all prescription-specific models but increased significantly for the mixed-prescription model, with approximately twofold higher errors and statistically significant deviations from the reference plans. Volumetric coverage followed a similar trend, with prescription-specific models yielding lower V_95%_ errors than the mixed-prescription model. Homogeneity index errors were comparable across all models and did not differ significantly from the reference plans. Altogether, seven of the eight metrics for Model 4 and three metrics for Model 3 deviated significantly from ground-truth distributions, whereas Model 1 showed no significant differences and Model 2 only two. These results indicate that prescription-dose stratification has its most pronounced effect on PTV coverage and hot-spot control, with mixed prescriptions leading to clearly larger PTV errors.

**Table 2:** Mean absolute error for the prediction of planning target volume by each model.

Matrices	Mean ± SD			
	Model 1 (50 Gy)	Model 2 (54 Gy)	Model 3 (60 Gy)	Model 4 (50 & 60 Gy)
D_99%_ (Gy)	3.08 ± 2.72	3.21 ± 3.06	4.01 ± 3.37*	3.62 ± 3.36
D_98%_ (Gy)	2.30 ± 2.46	2.53 ± 2.63	2.96 ± 3.09	3.47 ± 2.60*
D_95%_ (Gy)	1.87 ± 2.10	1.96 ± 2.16	2.23 ± 2.37	2.73 ± 1.81*
D_mean_ (Gy)	0.48 ± 0.49	0.52 ± 0.35	0.58 ± 0.49	1.27 ± 0.57*
D_2cc_ (Gy)	0.49 ± 0.54	0.54 ± 0.40*	0.57 ± 0.48	1.27 ± 0.58*
D_5cc_ (Gy)	0.74 ± 0.88	0.75 ± 0.74	0.83 ± 0.90	1.44 ± 0.48*
V_95%_ (%)	4.68 ± 5.44	4.01 ± 5.01*	4.87 ± 5.89	7.70 ± 4.20*
HI	0.09 ± 0.08	0.09 ± 0.09	0.09 ± 0.08	0.09 ± 0.10

*Indicates a statistically significant difference between predicted and reference values (*p* < 0.05)

### Prediction accuracy for OAR metrics

**Fig. 2:**
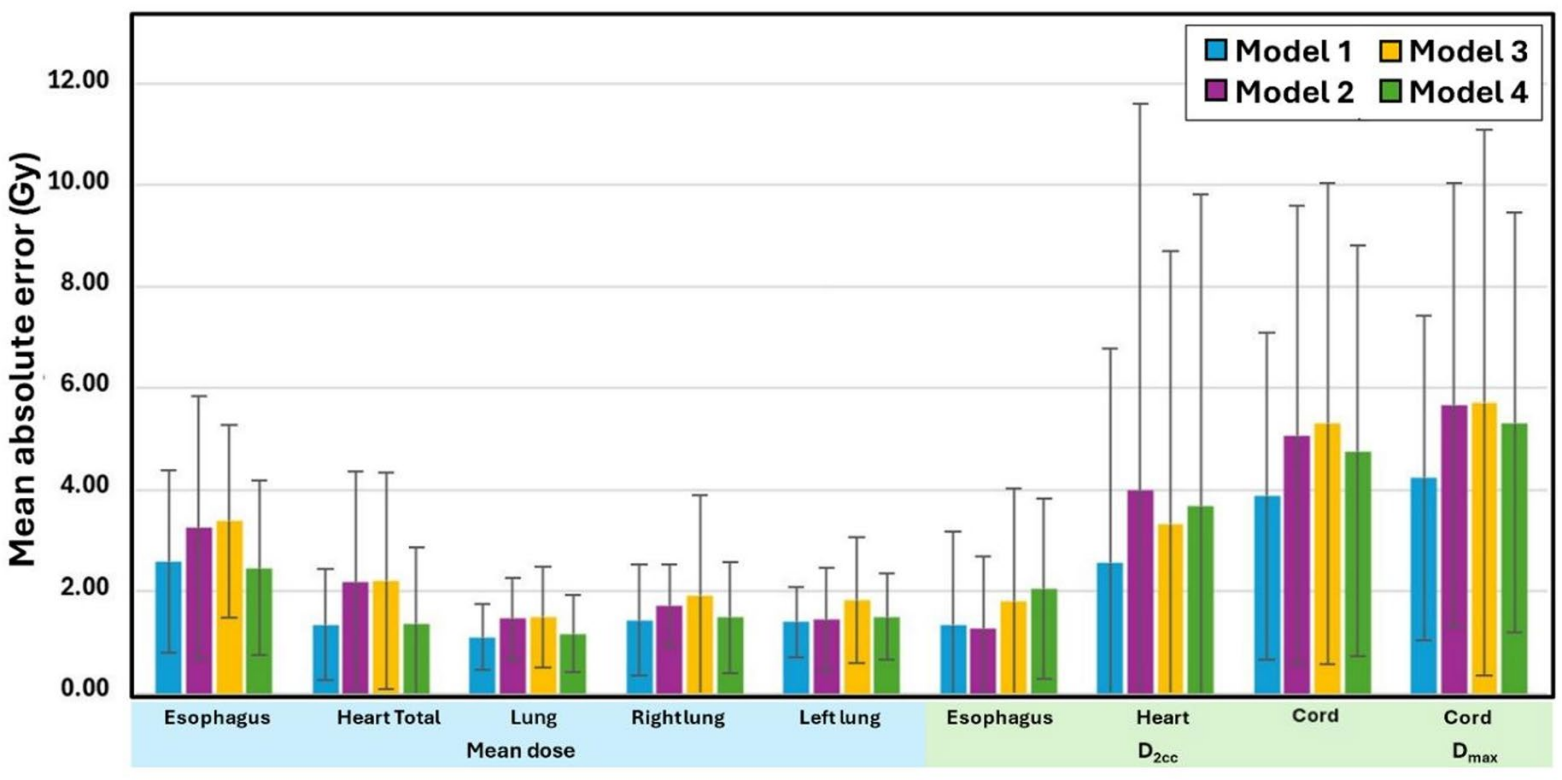
Mean absolute errors for the esophagus, heart, and lungs across four models, evaluated using dose-based metrics (mean dose, D_max_, and D_2cc_).

**Fig. 3:**
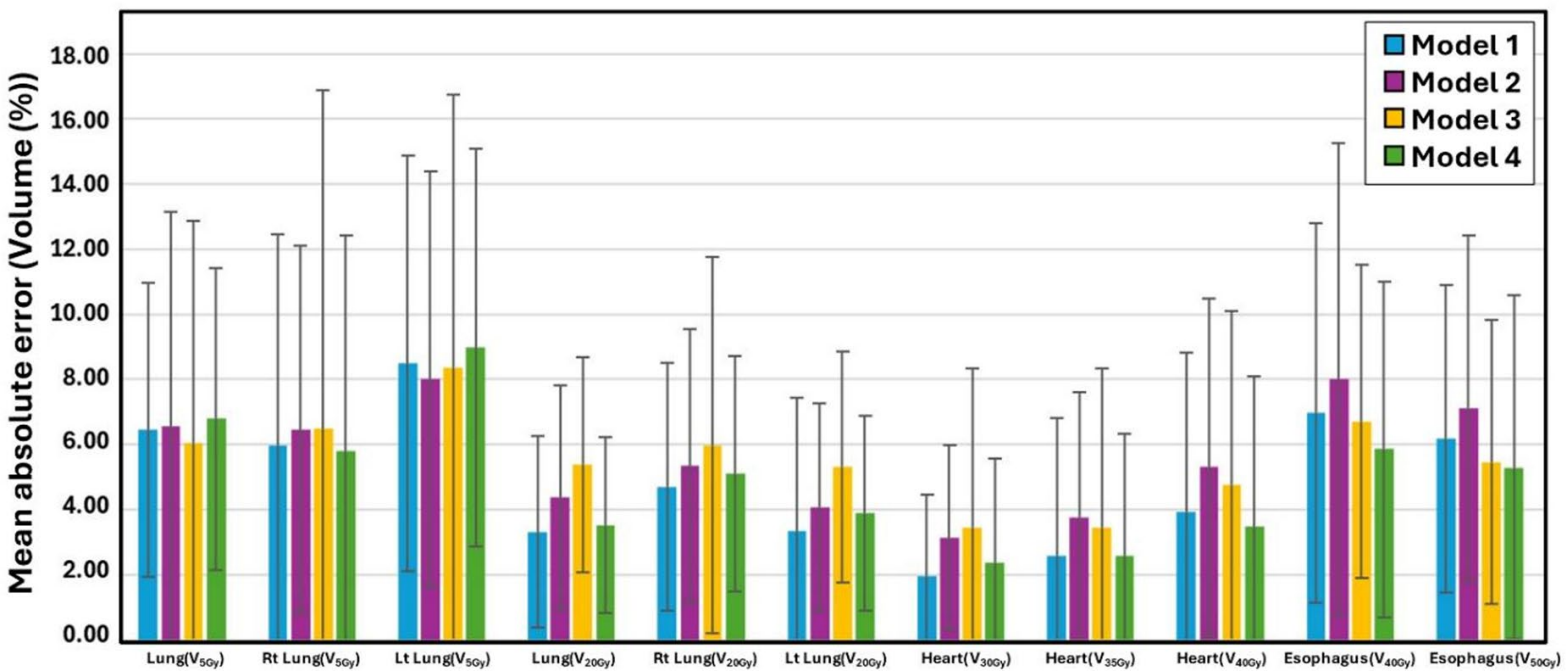
Mean absolute errors for the esophagus, heart, and lungs across four models, evaluated using volume-based dose metrics (V_5Gy_, V_20Gy_, V_30Gy_, V_35Gy_, V_45Gy_, and V_50Gy_), where V_xGy_ denotes the volume of the organ-at-risk receiving at least x Gy.

Figures 2 and 3 present the MAE values for all OAR dose-volume parameters across the four models, and detailed numerical values for each OAR metric are reported in Supplementary Table S1. For the esophagus, differences among the 50 Gy, 54 Gy, 60 Gy, and mixed-prescription models were small and never reached statistical significance. Mean-dose errors stayed between 2.46 ± 1.72 Gy and 3.39 ± 1.90 Gy, while the maximum dose of 2 cm³ (D_2cc_) varied from 1.27 ± 1.42 Gy to 2.06 ± 1.77 Gy. Volume-based errors at 40 Gy and 50 Gy did not exceed 8% for any model. Cardiac predictions showed a similarly narrow spread. Model 1 gave a heart mean-dose error of 1.35 ± 1.10 Gy; Models 2, 3, and 4 produced values up to 2.22 ± 2.13 Gy, but none differed significantly. D_2cc_ errors ranged from 2.57 ± 4.21 Gy to 4.00 ± 7.60 Gy, again without statistically significant variation. For the heart, volume‑based mean absolute errors remained modest across all models: V_30Gy_ MAEs were 1.95 ± 2.49%, 3.14 ± 2.85%, 3.45 ± 4.87%, and 2.37 ± 3.19% for Models 1–4, V_35Gy_ MAEs were 2.56 ± 4.25%, 3.74 ± 3.86%, 3.45 ± 4.87%, and 2.56 ± 3.77%, and V_40Gy_ MAEs were 3.92 ± 4.88%, 5.31 ± 5.18%, 4.75 ± 5.35%, and 3.46 ± 4.62%, respectively. Mean‑lung‑dose errors were modest—1.10 ± 0.65 Gy to 1.50 ± 1.00 Gy for the combined lungs and 1.40 ± 0.70 Gy to 1.92 ± 1.97 Gy for each lung—while low‑ and mid‑dose volume errors remained small (combined‑lung V_5Gy_ MAEs 6.04–6.79%, V_20Gy_ MAEs 3.30–5.38%; right‑lung V_5Gy_ 5.79–6.49%, V_20Gy_ 4.69–5.98%; left‑lung V_5Gy_ 8.00–8.98%, V_20Gy_ 3.32–5.31%). The spinal cord was the only structure for which model choice proved significant. Compared with Model 1, Models 2, 3, and 4 produced larger MAEs in D_2cc_—about 5 Gy (*p* = 0.013 for Model 2; *p* = 0.010 for Model 4)—and in D_max_, which reached 5.68–5.72 Gy (*p* ≤ 0.049). Model 1 showed lower values—3.88 ± 3.22 Gy for D_2cc_ and 4.24 ± 3.19 Gy for D_max_—which were not statistically significant (*p* > 0.05). No other OAR metric displayed significant inter-model variation. Taken together, these findings suggest that most OAR metrics are relatively robust to prescription mixing, with the spinal cord being the main exception and the most prescription-sensitive OAR to prescription-dose stratification.

### Dose distribution and DVH-based assessment

**Fig. 4:**
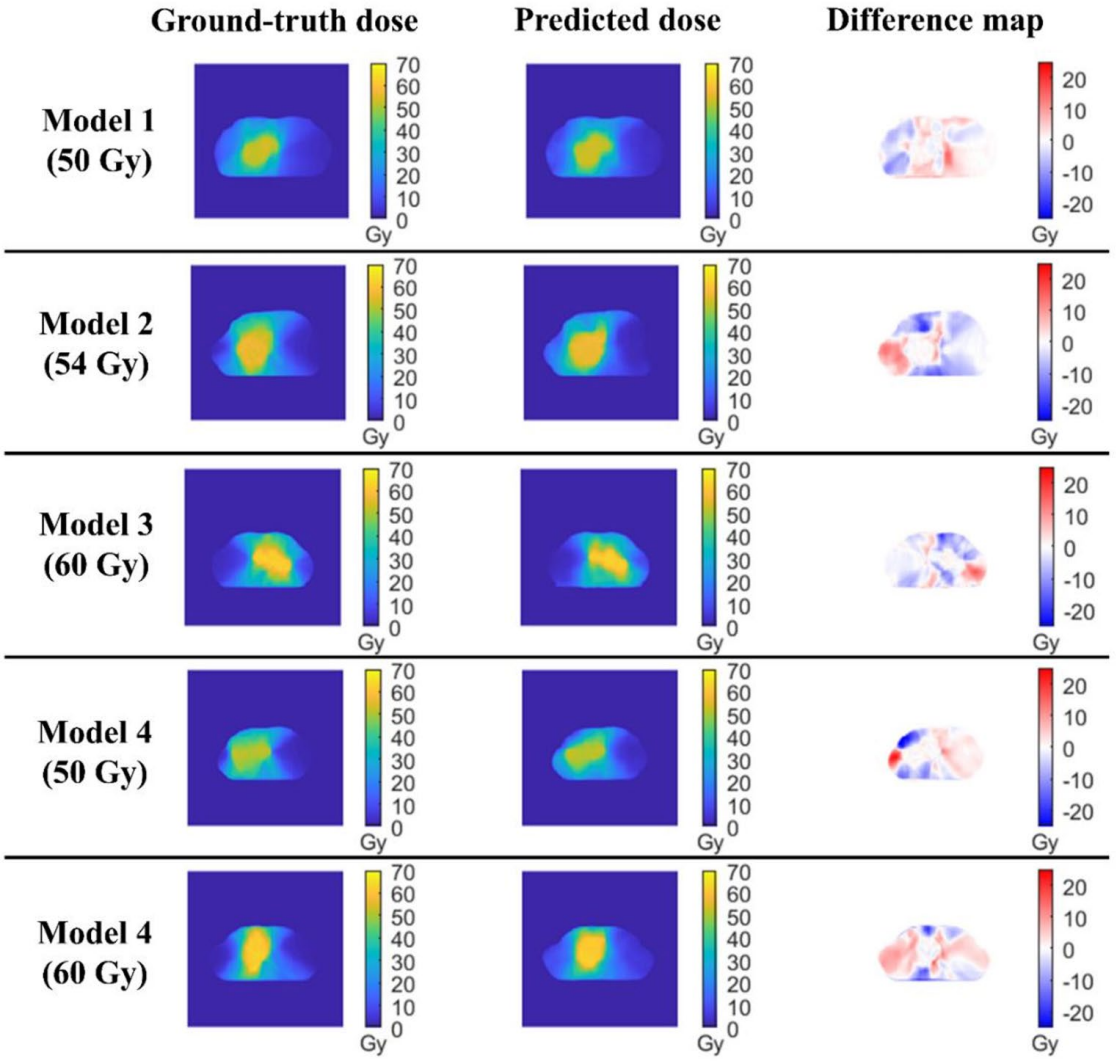
Example axial dose distributions for four models (Model 1: 50 Gy; Model 2: 54 Gy; Model 3: 60 Gy; Model 4: mixed 50 + 60 Gy): ground truth, prediction, and signed dose difference (prediction − ground truth).

**Fig. 5:**
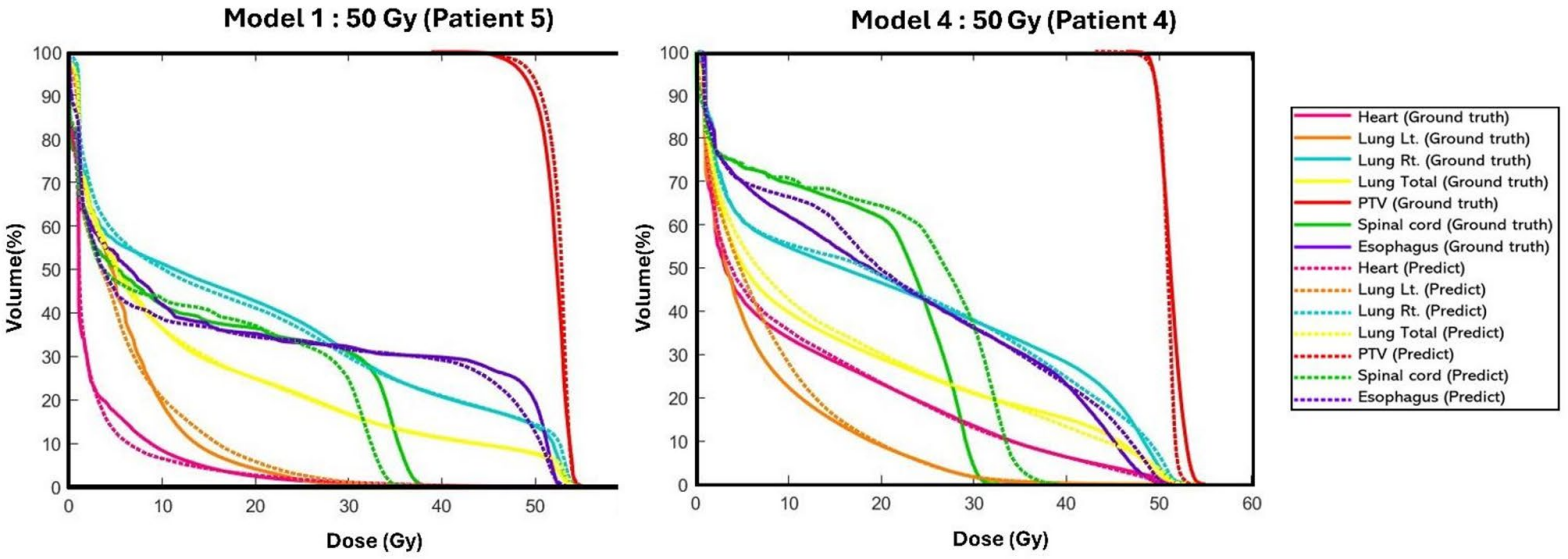
Dose–volume histograms for PTV and OARs for two representative 50 Gy test cases: Model 1 (Patient 5, left) and the mixed‑prescription Model 4 (Patient 4, right). Solid lines indicate ground truth and dashed lines the predicted DVHs. The Model‑4 panel illustrates a case with larger spinal cord deviations compared with the single‑prescription model.

Overall, the models preserved clinically meaningful DVH trends, but voxel-wise discrepancies persisted, most notably in the low-dose lung bath and in steep-gradient regions near small serial organs (spinal cord and, to a lesser extent, esophagus). Visual inspection (Fig. 4) showed good agreement of high-dose PTV regions across models, yet signed difference maps (prediction − ground truth) revealed localized deviations in the low-dose lung bath and near steep gradients around the spinal cord and esophagus regions. DVH comparisons (Fig. 5) were consistent overall: for a typical 50 Gy case from the single‑prescription model (Model 1, Patient 5), PTV curves closely overlapped the ground truth and OAR DVHs showed only slight overestimation of low‑dose lung volume (< 30 Gy; 1–3%) and smoothing of high‑dose gradients for the spinal cord and esophagus (> 20 Gy, divergences up to  5 Gy), whereas the 50 Gy example from the mixed‑prescription model (Model 4, Patient 4) illustrates a more challenging case with visibly larger spinal cord deviations.

Collectively, the models demonstrated robust accuracy in predicting PTV dose distributions with minimal variability. OAR dose predictions were generally reliable, but limitations persist in low-dose lung regions and steep-gradient structures (spinal cord, esophagus).

## Discussion

This study demonstrates that prescription-stratified 3D U-Net models can predict VMAT dose distributions for locally advanced NSCLC with promising agreement. Importantly, the primary contribution of this work is not architectural innovation but the systematic investigation of prescription-dose stratification as a modeling and training strategy for VMAT dose prediction. By holding the network architecture and training pipeline constant, we isolate the effect of prescription handling and demonstrate its clinical relevance for treatment-planning decision-support, including feasibility screening and optimization guidance. By using an identical network architecture across models, we specifically quantified the effect of prescription-dose stratification on performance and found that prescription handling is a major determinant of prediction accuracy in this VMAT setting. Our primary finding is that stratifying models by prescription dose yields superior accuracy for PTV coverage and hot-spot metrics compared to a single model trained on mixed prescriptions, while most OAR doses remain relatively similar across models except for the spinal cord, a small serial organ with steep dose gradients.

To place these findings in context, we interpret them relative to prior work on DL–based dose prediction. Knowledge-based planning has advanced markedly since machine-learning techniques were first applied to radiotherapy dose prediction, yet variability in tumor geometry, beam modulation, and prescription level continues to limit accuracy. DL models have begun to close this gap, but most investigations have focused on fixed-field IMRT—most notably by Jhanwar et al.^17^, Barragán-Montero et al.^23^, and Miao et al.^26^. Because VMAT delivers radiation through continuous arcs, it introduces steeper gradients and greater low-dose spread—characteristics that challenge dose prediction networks trained on more uniform beam arrangements. By developing and evaluating prescription-specific convolutional networks for VMAT in locally advanced NSCLC, this study extends the evidence base into a modality now preferred for thoracic planning.

Our findings both agree with and extend previous work on DL–based dose prediction. In fixed-field IMRT, Shao et al.^27^ demonstrated that multi-prescription learning can be successful when prescription information is explicitly encoded in the network architecture. In contrast, our mixed-prescription VMAT model performed poorly, indicating that the continuous arc modulation of VMAT introduces greater complexity and makes multi-prescription learning more challenging without explicit conditioning. At the same time, our results confirm the findings of Barragán-Montero et al.^23^ and Zhang et al.^24^, showing that prescription-specific models can achieve high accuracy, particularly for PTV coverage and high-dose regions. While Cao et al.^25^ extended VMAT dose prediction across a broad prescription range, their study did not systematically isolate the impact of mixed versus stratified prescriptions within a fixed architecture. By holding the network design constant, our study demonstrates that prescription-dose stratification itself is a key determinant of prediction performance in VMAT, especially for structures with steep dose gradients such as the spinal cord. It is important to emphasize that the inferior performance of the mixed-prescription model observed in this study does not imply that mixed-prescription deep-learning dose prediction is inherently limited. Prior work has shown that multi-prescription models can perform well when prescription information is explicitly encoded through network inputs, loss design, or physics-informed constraints. In contrast, our mixed-prescription VMAT model was trained without explicit prescription conditioning and relied solely on anatomical information to infer the intended dose level. As implemented, the mixed-prescription model learned from heterogeneous data at two discrete prescription levels and should not be interpreted as a continuous, prescription-interpolating predictor; evaluation of interpolation across unseen prescription levels was beyond the scope of this study. Taken together, these findings indicate that prescription-dose stratification and prescription-aware mixed-prescription modeling should be viewed as complementary strategies rather than competing paradigms.

In this study, we trained three single-prescription models (50 Gy, 54 Gy, and 60 Gy) and a fourth model that combined the 50 Gy and 60 Gy cases. We trained and evaluated the models using total physical dose because clinical plan evaluation and feasibility decisions—including prescription feasibility and OAR constraint compliance (e.g., lung V_20Gy_ and spinal cord D_max_)—are made in total-dose terms. In practice, if a higher prescription is predicted to be unlikely to meet key OAR constraints, the prescription level or planning strategy (e.g., beam arrangement or optimization priorities) may be adjusted early in the planning process. An important future direction is to train the model to predict dose per fraction (or dose normalized to prescription) and then scale to total dose; this may reduce prescription-related domain shift, but it requires explicit validation of spatial agreement—particularly in steep-gradient regions and near serial OARs. In our evaluation, the single-prescription models reproduced high-dose coverage for the PTV with MAEs below 4 Gy; however, these DVH-metric MAEs are not directly comparable to clinical audit tolerances, which assess local dose agreement (e.g., point-dose differences analysis) and include additional sources of uncertainty. In contrast, the mixed-prescription model produced larger discrepancies across almost every PTV metric. The degradation mirrors the domain-shift effect described by Shao et al.^27^, who noted similar losses when heterogeneous beam-angle protocols were combined without explicit conditioning. These findings confirm that a network must learn two distinct dose-gradient regimes when prescriptions differ by ≥ 10 Gy; either stratification or a prescription-aware architecture is therefore essential.

For OARs, however, the choice of model was less critical. Mean-dose errors for the esophagus, heart, and lungs stayed below 3.5 Gy, and volume-based MAEs remained under 8% regardless of the model. Because the dose to these large, low-contrast structures is governed mainly by patient anatomy and global beam geometry, the model generalized across prescriptions without appreciable loss of fidelity. The spinal cord was the exception. Its small cross-section and steep surrounding gradients magnify spatial misalignments; once high-prescription cases were introduced, MAEs for both D_2cc_ and D_max_ rose to ≈ 5–6 Gy and became statistically significant—roughly 10% of the customary 45 Gy tolerance. Consistent with findings by Barragán-Montero et al.^23^ and by Shao et al.^27^, these results suggest that serial organs with sharp gradients benefit from prescription-specific models or loss functions that assign greater weight to high-dose voxels.

Our results compare favorably with earlier IMRT studies. Despite the added complexity of continuous-arc delivery, the 60 Gy model achieved PTV MAEs that differ from those reported by Barragán-Montero et al.^23^ by < 1 Gy for D_99%_, D_98%_, D_95%_, and D_5cc_. Likewise, the mixed network outperformed Shao et al.^27^ for all core PTV metrics at both 50 Gy and 60 Gy prescriptions, and lung mean-dose predictions differed from prior benchmarks by ≤ 0.3 Gy. Slight overestimation of esophageal V_40Gy_ and V_50Gy_ (≈ 1–2% volume) and higher variability in heart V_40Gy_ mirror the challenges others have reported in modeling low-dose bath—an area influenced by tumor location and breathing-induced anatomic changes.

Visual inspection of axial dose distributions (Fig. 4) showed good alignment of the high-dose isodose contours between the predictions and ground truth across all models. The difference maps are displayed with a 20 Gy color scale to show the full dynamic range; however, within the PTV and OARs, most voxels differed by only a few Gy. The largest deviations were rare and occurred predominantly at the periphery of high-dose regions and in voxels outside the contoured structures used for training, where steep dose gradients make the prediction problem more challenging. These regions have minimal influence on the DVH-based metrics reported in Table 2 and S1. It is also important to clarify the rationale behind our choice of evaluation metrics. We focused on MAE and DVH-derived parameters because they provide direct, clinically interpretable measures of dose accuracy. Other similarity metrics, such as the Gamma index and Pearson correlation coefficient, were not used for methodological reasons. Gamma analysis is highly sensitive to spatial resolution and interpolation, and our model outputs were generated on resampled 128 × 128 × 128 grids, which might yield gamma results that do not reflect true clinical discrepancies. Similarly, correlation coefficients capture global agreement but may miss clinically relevant local deviations in steep dose-gradient regions. For these reasons, MAE and DVH metrics were considered more appropriate for evaluating clinically meaningful dose differences, while future studies may incorporate gamma analysis on native TPS grids and correlation-based measures as complementary tools. Although DVH-based errors were generally modest, signed difference maps revealed localized deviations—particularly in the low-dose lung bath and in high-gradient regions near small serial organs (e.g., spinal cord). These discrepancies are important because they can be clinically relevant even when summary DVH metrics appear acceptable, and they limit the current framework to planning guidance and benchmarking rather than dosimetric verification.

The proposed framework is intended as a treatment planning decision-support and benchmarking tool rather than a replacement for the TPS dose calculation. In practice, the predicted dose distribution and derived DVH metrics may be used for early feasibility screening (e.g., identifying cases likely to violate key OAR constraints before time-consuming optimization) and for optimization guidance by providing a patient-specific reference dose pattern that can inform inverse planning or automated planning pipelines. Given the observed voxel-wise deviations, particularly in low-dose lung regions and near steep gradients, the model output should be interpreted as a planning aid; final dose computation, clinical approval, and patient-specific QA remain within the standard TPS-based workflow.

Several limitations and future research directions should be considered. First, GPU‑memory‑restricted input size and the required down‑sampling may blunt spatial fidelity, particularly in low-dose volumes such as lung V_5Gy_ and V_20Gy_, and could impact normal tissue complication probability assessments. These deviations suggest the need for improved model calibration, as minor overestimations in esophagus V_40Gy_ and V_50Gy_ could influence toxicity predictions and dose constraints. Similarly, higher variability in heart V_40Gy_ predictions could necessitate additional safety margins to mitigate cardiac toxicity risks. Second, although 72 cases provide more heterogeneity than many single-center series, the dataset remains modest and increases the risk of over-fitting. Third, the spinal cord sample contained relatively few voxels near the tolerance threshold, which likely contributed to its larger prediction variance. Lastly, the impact of the dose calculation grid size on model performance warrants further investigation. While we maintained a uniform 2.5 mm grid resolution, clinical practice may involve different grid sizes, and model robustness to such variations should be evaluated. Taken together, these findings underscore that prescription-dose stratification is not a minor implementation detail but a key modeling decision that directly affects clinically relevant prediction accuracy, especially for PTV coverage and spinal cord sparing. Future work will therefore focus on prescription‑aware mixed‑prescription modeling, including explicit prescription or fraction‑number inputs, prescription‑normalized outputs, and gradient‑ or organ‑at‑risk–weighted loss functions to mitigate the domain shift observed in the current anatomy‑only implementation. An additional limitation is the modest size and single‑institution origin of the cohort, combined with a data‑splitting strategy that relies heavily on left–right flipping within the training set while reserving 10 and 20 completely independent patients for validation and testing, respectively. Although this approach avoids patient‑level data leakage between splits, it reduces the diversity of non‑mirrored anatomies available for training and may lead to somewhat optimistic validation performance, particularly for left–right asymmetries that are not fully captured by flipping. In future work, larger multi‑institutional datasets, alternative splitting schemes such as cross‑validation, and more diverse augmentation (e.g., elastic deformations, intensity perturbations) will be important to improve robustness and generalizability.

In addition, this study includes explicit binary masks of the PTV and multiple OARs as input channels to enable anatomy‑aware learning of clinically relevant trade‑offs between target coverage and OAR sparing, which are critical to prescription‑feasibility assessment and optimization guidance. A potential trade‑off of using multiple OAR masks is increased input dimensionality and data requirements; however, this is offset by clearer encoding of serial versus parallel organ geometry, which is essential for constraint‑focused feasibility assessment in thoracic VMAT. This design follows common practice in DL‑based dose prediction, where CT images and multiple structure masks (PTV and OARs) are provided as multi‑channel inputs to encode anatomical context for voxel‑level dose estimation^17,20,22–25^. This design contrasts with recent work by Loebner et al. (DeepSMCP)^28^, who proposed a deep‑learning–based denoising framework that uses a fast, high‑statistical‑uncertainty Monte Carlo dose distribution together with CT as inputs—without explicit OAR masks—to recover a low‑noise Monte Carlo dose distribution at substantially reduced computational time. While DeepSMCP focuses on accelerating high‑fidelity dose calculation for a given treatment plan and beam configuration, our framework targets an earlier stage of the workflow by predicting anatomy‑conditioned dose patterns for prescription feasibility screening and VMAT optimization guidance^20,24,25^. These approaches are therefore complementary rather than competing: DeepSMCP refines a physics‑based approximate dose, whereas our model provides an anatomy‑based prior that can inform planning decisions before final dose computation, and a promising future extension would be to combine anatomical masks with fast, approximate dose inputs (e.g. coarse or noisy Monte Carlo) to further reduce local discrepancies, particularly in steep‑gradient regions and near serial organs^24,25^.

In conclusion, these findings highlight key directions for improving clinical applicability. These limitations identify specific areas for improvement to enhance clinical applicability: (1) increasing cohort size and diversity (ideally multi-institutional) to improve generalizability; (2) incorporating explicit prescription and/or fractionation conditioning to reduce domain shift; (3) using higher-resolution or multi-scale modeling and gradient-aware loss functions to better capture steep dose fall-off near serial organs; and (4) adding uncertainty estimation to flag cases or regions where predictions are less reliable and warrant closer review.

## Conclusion

This study evaluated the impact of prescription-dose stratification on DL–based VMAT dose prediction for NSCLC. Prescription-stratified 3D U-Net models trained on individual prescription levels (50, 54, and 60 Gy) were able to closely reproduce the corresponding clinical dose distributions, with the most pronounced benefit observed for PTV coverage, where mean absolute errors remained below 4 Gy and OAR dose errors were low for most organs. In contrast, a mixed-prescription model trained on 50 and 60 Gy plans degraded performance, increasing PTV coverage and hot-spot errors, while doses to the lungs, heart, and esophagus changed only modestly across models. These findings indicate that prescription-dose stratification is a key design choice for reliable VMAT dose prediction, especially for ensuring adequate PTV coverage, and suggest that such models can serve as robust clinical decision-support tools to improve treatment planning quality and efficiency. They should be validated further in larger, multi-institutional cohorts before routine clinical implementation.

## Supplementary Information

Below is the link to the electronic supplementary material.

Supplementary Material 1

## Data Availability

The datasets generated and/or analyzed during the current study are available from the corresponding author on reasonable request.
