# Supplementary material for: Prescription‑dose stratification improves deep learning‑based VMAT dose prediction in locally advanced NSCLC
Source: Sci Rep. 2026 Mar 9;16:8707. doi: 10.1038/s41598-026-43192-6 (PMC12979670; doi:10.1038/s41598-026-43192-6)
Supplement: Supplementary file 1 — Supplementary Material 1 [file 41598_2026_43192_MOESM1_ESM.docx]

# Supplementary Table S1

Mean absolute error (MAE ± SD) for organ-at-risk (OAR) dose–volume metrics across the four models.

| **Organ** | **Criteria** | **Mean ± SD** | | | |
| --- | --- | --- | --- | --- | --- |
|  |  | **Model 1 (50 Gy)** | **Model 2 (54 Gy)** | **Model 3 (60 Gy)** | **Model 4 (50 & 60 Gy)** |
| Esophagus | Mean | 2.59 ± 1.79 | 3.25 ± 2.60 | 3.39 ± 1.90 | 2.46 ± 1.72 |
|  | D_2cc_ | 1.34 ± 1.85 | 1.27 ± 1.42 | 1.80 ± 2.24 | 2.06 ± 1.77 |
|  | V_40Gy_ | 6.96 ± 5.84 | 8.00 ± 7.26 | 6.70 ± 4.81 | 5.85 ± 5.16 |
|  | V_50Gy_ | 6.16 ± 4.73 | 7.11 ± 5.31 | 5.46 ± 4.37 | 5.29 ± 5.28 |
| Heart | V_30Gy_ | 1.95 ± 2.49 | 3.14 ± 2.85 | 3.45 ± 4.87 | 2.37 ± 3.19 |
|  | V_35Gy_ | 2.56 ± 4.25 | 3.74 ± 3.86 | 3.45 ± 4.87 | 2.56 ± 3.77 |
|  | V_40Gy_ | 3.92 ± 4.88 | 5.31 ± 5.18 | 4.75 ± 5.35 | 3.46 ± 4.62 |
|  | Mean | 1.35 ± 1.10 | 2.19 ± 2.18 | 2.22 ± 2.13 | 1.36 ± 1.52 |
|  | D_2cc_ | 2.57 ± 4.21 | 4.00 ± 7.60 | 3.33 ± 5.37 | 3.69 ± 6.12 |
| Spinal cord | D_2cc_ | 3.88 ± 3.22 | 5.08 ± 4.51* | 5.31 ± 4.74 | 4.76 ± 4.04* |
|  | D_max_ | 4.24 ± 3.19 | 5.68 ± 4.37* | 5.72 ± 5.37* | 5.32 ± 4.13* |
| Lung (Both) | Mean | 1.10 ± 0.65 | 1.47 ± 0.80 | 1.50 ± 1.00 | 1.17 ± 0.76 |
|  | V_5Gy_ | 6.46 ± 4.52 | 6.56 ± 6.58 | 6.04 ± 6.82 | 6.79 ± 4.64 |
|  | V_20Gy_ | 3.30 ± 2.94 | 4.36 ± 3.45 | 5.38 ± 3.30 | 3.51 ± 2.70 |
| Right Lung | Mean | 1.44 ± 1.09 | 1.73 ± 0.80 | 1.92 ± 1.97 | 1.49 ± 1.09 |
|  | V_5Gy_ | 5.98 ± 6.48 | 6.46 ± 5.64 | 6.49 ± 10.39 | 5.79 ± 6.64 |
|  | V_20Gy_ | 4.69 ± 3.80 | 5.33 ± 4.20 | 5.98 ± 5.79 | 5.10 ± 3.62 |
| Left Lung | Mean | 1.40 ± 0.70 | 1.45 ± 1.01 | 1.83 ± 1.24 | 1.50 ± 0.85 |
|  | V_5Gy_ | 8.49 ± 6.40 | 8.00 ± 6.38 | 8.36 ± 8.38 | 8.98 ± 6.11 |
|  | V_20Gy_ | 3.32 ± 4.12 | 4.07 ± 3.18 | 5.31 ± 3.55 | 3.89 ± 2.99 |

* Indicates a statistically significant difference between predicted and reference values (p < 0.05).
